# A comparative analysis of syntactic complexity in English academic texts by Chinese MTI trainee translators and native academic writers

**DOI:** 10.1371/journal.pone.0357458

**Published:** 2026-09-08

**Authors:** Jianqi Guo, Yingmei Qu

**Affiliations:** 1 School of International Studies, Northeast Normal University, Jilin, China; 2 School of International Studies, Northeast Normal University, Jilin, China; Shanghai International Studies University, CHINA

## Abstract

This paper aims to investigate the differences in syntactic complexity between academic English translation by Chinese MTI trainee translators (TTAE) and academic English by native writers (NAE). The study is based on two self-built corpora and employs the L2 Syntactic Complexity Analyzer (L2SCA) to analyze syntactic complexity. The results show that the two groups of texts differ significantly in nine out of fourteen measures of syntactic complexity. The syntactic complexity of NAE is higher in the T-unit complexity ratio, Complex T-unit ratio, Dependent clause ratio, and Dependent clause per T-unit, while the syntactic complexity of TTAE is higher in Complex nominals per T-unit, Complex nominals per clause, Coordinate phrases per T-unit, Coordinate phrases per clause, and Mean length of clause. Compared with native writers who tend to use shorter clauses, more subordinate structures, and more complex T-units, Chinese MTI trainee translators produce longer clauses, coordinated structures, complex noun phrases, and relatively reduced syntactic dispersion. Those findings suggest a syntactic profile that favors phrasal elaboration over clausal elaboration, accompanied by relatively reduced syntactic complexity. The reasons for the differences are briefly discussed, revealing the influence of L2 language development, source -text effect, and translation strategies. This research provides insights for translation teaching and contributes to a better understanding of the syntactic characteristics of trainee translators’ academic English.

## Introduction

Translators’ syntactic competence has a direct impact on their ability to understand the source language and their fluency and readability of the target language, which reflects the ability of language learners to construct complex discourse [1,2]. Syntactic complexity, as an important indicator of syntactic competence and linguistic proficiency, is therefore central to the evaluation of the translation outcome of trainee translators. While existing research has examined syntactic complexity in L2 writings, the situation in translation texts, especially those produced by trainee translators, remains underexplored. As translation competence is regarded qualitatively different from the bilingual competence [3], trainee translators constitute a distinctive group of second language users. Examining their syntactic performance therefore extends the current research beyond the general L2 writing by enriching the description of the syntactic complexity of non-native English

Native academic writers have traditionally been adopted as a reference group in cross-linguistic studies for comparative purpose. Although existing research has extensively compared the syntactic complexity of native and second-language writing [4,5], very little is known about the systematic differences between translation and native academic writing [6], let alone the comparison involving translation by trainee translators and their native academic counterparts. Addressing the research gap mention above,the present research aims to identify and analyze the key linguistic differences in syntactic complexity between academic English translation texts by Chinese MTI trainee translators (TTAE) and academic English texts by native writers (NAE). Such comparison is both theoretically and empirically important. It aims to identify the distinctive syntactic features that characterize TTAE, and to offer valuable insights for improving the training of trainee translators, helping them better adapt to the demands of the academic translation market.

The remainder of the paper is organized as follows.Section 1 reviews the relevant literature, followed by research questions and methodology in Section 2 and section 3. Section 4 presents the results and comparative analysis of syntactic complexity between TTAE and NAE. Section 5 discusses the main findings Section 6 concludes the paper.

## Section1: Literature review

### Syntactic complexity in academic discourses

According to Ortega, syntactic complexity refers to the range of forms that surface in language production and the degree of sophistication of such forms [7]. Previous studies on syntactic complexity have mainly focused on writing or speaking English as a foreign language. Many studies have analyzed writings produced by L2 learners at different proficiency levels, finding that syntactic and phrasal complexity generally increases as language proficiency improves [4,8–12]. Some have compared the syntactic complexity of L2 learners with that of native speakers and found that there are great differences between the syntactic complexity of texts written by L2 learners and native speakers [6,13]. Based on their research, non-native writers tend to use shorter, simpler sentence structures and employ subordinate structures less frequently than native writers. Kuiken and Vedder document variations in the development of syntactic complexity across proficiency levels, across languages, and between L2 and L1 writing [14].

The syntactic complexity in academic discourses has drawn much attention from scholars. Some research compares L1 and L2 academic writers and finds that EFL writers’ research articles differ significantly from comparable native-language research articles, among whom L1 Chinese writers prefer complex nominals [5,15, 16]. Research on the differences in syntactic complexity between emerging and expert academic writers shows that emerging authors’ academic discourse is less consistent in their syntactic structure choices to achieve the communicative functions [17]. And there are significant differences in syntactic complexity among academic texts from different disciplines [16,18,19]. Scholars also interviewed students and found that their academic writing benefited from targeted instruction of syntactic complexity [20].

Many factors contribute to the syntactic complexity in academic discourse. Syntactic complexity can be attributed to linguistic features of the given language(s), text genre, and the particular author’s (idiosyncratic) lexical and grammatical preferences [21]. It is also influenced by the task type, learners’ pragmatic performances, and even temporal changes [22–24]. Descriptive and narrative texts tend to have lower syntactic complexity, while expository and argumentative texts display higher levels of complexity [9,12,17,25]. Yin Shuhui, Gao Yuan, and Lu Xiaofei also found that the syntactic complexity of emerging Chinese international publication writers is related to their rhetorical strategies, which are influenced to some extent by editors and reviewers [26].

### Automated tools for Syntactic complexity analysis

The conceptualization of syntactic complexity, together with the development of syntactic complexity analyzers, has significantly influenced research on syntactic complexity in L2 writing assessment. Wolfe-Quintero et al. classified the measures in studies of second language development into fluency, accuracy and complexity (both grammatical and lexical), with syntactic complexity as the key indicator of linguistic development [8]. Lu further summarized these measures and developed the L2 Syntactic Complexity Analyzer (L2SCA) which has been widely used, featuring as saving time, standardizing the measures, and expanding the sample size in syntactic complexity research [9]. At present, the commonly used automatic syntactic complexity analyzers are Biber Tagger, Coh-Metrix, L2SCA, and TAASSC [27–30]. They differ in analytical focus. Among these tools, the Biber Tagger provides foundational tagging for register-based analysis, Coh-Metrix assesses syntactic complexity through parse-tree representations of sentence structure, and L2SCA offers a set of 14 theoretically motivated measures specifically designed for L2 writing [31]. TAASSC, on the other hand, automatically identifies and quantifies both macro-level and micro-level syntactic complexity features in writing and offers a more extensive set of indices than L2SCA [4]. Polio and Yoon tested the validity of SCA and Coh‐Metrix and found that both of them are valid for studying variation in complexity [25]. Several new analyzers have also emerged, such as the corrected type-token ratio (TTR) of dependency relations introduced by Bi and Jiang [32].

This study adopts L2SCA for its analysis, as its metric system has been extensively validated and widely used in the field of second language acquisition, ensuring that the results possess strong comparability and explanatory power.

In summary, syntactic complexity has been extensively studied in L2 writing, especially academic writing, and supported by a wide range of analytical tools. However, the research on syntactic complexity in translation, as an important aspect of L2 use, remains comparatively underexplored. In the next section, we will discuss the syntactic complexity in translation text.

### Syntactic complexity in translation text

The research on Syntactic complexity has extended from EFL writing texts into translation texts. Translation entails producing a text for the target context, taking the target purpose and the target audience into consideration. Different from L2 writing, translation involves transforming the content of the source language into the target language, and the processing of two languages simultaneously, which entails a complex cognitive load and may affect the complexity of linguistic form. Uzawa compared L2 learners’ writing and translation processes across three tasks and found that the development modes are different between writing and translation processes [33]. Additionally, patterns of attention allocation and attention to language use differ significantly between the two tasks. Thus, it’s necessary to explore the profile of syntactic complexity in translation texts.

Syntactic complexity plays an important role in translation. It affects the communicative effect of the text, thereby affecting the quality of translation.Over-simplified syntax may lead to a loss of information or a change of meaning, while the over-complex syntax may affect the clarity of the translation text [2]. Language-specific syntactic differences between L1 and L2, such as the differences in direction of branching and noun modifiers between English and Chinese, can also significantly impact the accuracy of translation [34]. Syntactic complexity is even related to the realm of translation style [35].

Syntactic complexity has been applied to improve translation quality and advance AI-related research. Scholars have proposed several measurements, such as dependency syntax and cross-linguistic alignment [36,37]. Moreover, some scholars are taking machine translation and GenAI into consideration. Kwok et al. discovered that GPT post-editing improved trainee translators’ lexical complexity, but its effect on syntactic complexity was inconsistent [38]. Saeed also discovered that Google Translate output has lower syntactic complexity ratios for most of the 10 indices measured [39].

Previous research has examined syntactic complexity by comparing translated English with non-translated English across different genres and contexts [40,41]. Some studies have proved that compared to non-translation, translation texts are generally less complex [6,42–45]. However, other studies have produced contrasting findings, showing that, in certain cases, the syntactic complexity of translated English exceeds that of native English writing [40,42]. Wang et al. discovered that there are no significant differences between the two [46]. In addition, Tan observed that, compared with native academic English, Chinese-to-English academic expert-translation texts exhibit both signs of simplification and signs of increased complexity in different measures [47]. These findings suggest that the syntactic complexity differences between translated English and native academic English are not simply a matter of being more or less complex overall; rather, they may involve more nuanced, specific variations.

Although previous research has recognized the importance of studying syntactic complexity in translated texts, existing research has primarily focused on expert translators, with relatively limited attention given to trainee translators. The trainee translator is a product of the mid-twentieth-century institutionalization of translator training, which transformed entry into the profession from informal pathways to a globally expanding system of formal education [48]. Existing research on trainee translators has primarily addressed issues concerning training methods [49–53], translation process and recognition features [54–59], the application of translation tools including the Internet and AI [60,61], and so forth. Trainee translators’ productions are constrained at both the macro and micro levels through factors like translation experiences, idiosyncrasies, and source text features [62]. Studies examining linguistic features of trainee translators’ output remain relatively limited. The available research mainly focus on high-frequency lexical bundles, source-text comprehension, terminology management, rhetorical expression, and translation errors [63–65]. Compared with these aspects, syntactic complexity has received considerably less attention, despite its importance as an indicator of academic writing development.

Given that native academic writing is commonly adopted as a reference in research on L2 academic writing, comparing TTAE with NAE provides an opportunity to characterize the syntactic complexity profile of trainee translators’ translated academic texts. However, research comparing the syntactic complexity of academic English translation texts produced by trainee translators with native academic English remains scarce. It is still unclear to what extent trainee translators’ translated texts resemble or differ from native academic writings across different dimensions of syntactic complexity.

Addressing these gaps, the present research compares trainee translators’ academic English translation (TTAE) with native writers’ academic English (NAE) to characterize the syntactic complexity profiles of academic translation by trainee translators.

## Section2: Research questions

This paper conducts an empirical study by employing a corpus-based approach to analyze the syntactic complexity between TTAE and NAE. To systematically discern the differences, both the common syntactic patterns shared by these two text types and the distinctive patterns unique to each variety are examined, thereby providing pedagogical insights for the training of trainee translators.

More precisely, the following research questions will be explored:

RQ1: What are the significant differences in syntactic complexity between TTAE and NAE?

RQ2: What specific features of TTAE’s syntactic complexity are revealed in comparison with the syntactic complexity of NAE?

## Section 3: Methodology

### Data selection

To ensure the validity of the comparative study, this research adhered to the principle of comparability when constructing the TTAE corpus and the NAE corpus. The descriptive statistics of the two corpora are presented in Table 1. The two corpora are highly consistent in scale. Both TTAE (academic English translation by trainee translators) and NAE (academic English texts by native writers) contain 38 texts, with a total of 381,548 and 381,645 tokens respectively, and mean text lengths of 10,040.74 and 10,043.29 tokens. Such equivalence in text number and length provides a solid basis for subsequent comparative analysis of syntactic features.

**Table 1:** Descriptive statistics of TTAE and NAE.

Corpus	Text count	Tokens	Mean size
TTAE	38	381,548	10040.74
NAE	38	381,645	10043.29

The TTAE corpus comprises English translation texts produced by 38 third-year Chinese students enrolled in a Master of Translation and Interpreting (MTI) program. Introduced in 2007, MTI is a professional master’s degree to provide systematic and professional translation training [66,68]. Chinese MTI students can be regarded as an appropriate sample of trainee translators. Based on more than two years of translation training and practice, these students demonstrated a relatively high level of bilingual proficiency, and all possessed a foundation in academic translation. The Chinese MTI trainee translators are from 26 universities in China. To ensure the quality of the corpus, all translations were reviewed and approved by the trainees’ supervisors before being included in the analysis.

The NAE corpus consists of excerpts from 38 academic monographs written by different native English writers published by internationally renowned publishers and prestigious university presses. These texts represent the high-level writing paradigms of English academic discourse in the relevant fields. We also checked the lingusitic background of these scholars to ensure they are native English writers. The monographs in NAE cover different publication periods, with most published between 2010 and 2020, and are authored by native English scholars primarily from the United States, the United Kingdo, and other English-speaking countries.

Accordingly, this study analyzes selected excerpts from monographs by trainee translators and native academic writers. To ensure comparability, we matched the Chinese source texts and native English academic English texts on several indices, including genre, disciplinary coverage, corpus size, and text length, and Table 2 is the brief information of source texts of TTAE and NAE.

**Table 2:** Descriptive statistics of source texts of TTAE and NAE.

	Text Count	Genre	Disciplinary Coverage	Tokens	Mean Size
NAE	38	academic monograph	8	381,645	10,043.29
Source Texts of TTAE	38	academic monograph	8	347,284	9,139.05

Genre and disciplinary interference are avoided by ensuring a balanced distribution across subject areas. The texts in both corpora are confined to the domain of social science, precisely covering eight sub-disciplines: geography, economics, history, literature, linguistics, philosophy, sociology, and education. Because each corpus contained 38 texts, every sub-discipline was represented by four or five texts. The same disciplinary distribution was maintained across the two corpora to ensure corpus comparability and to minimizing the potential impact of disciplinary differences on syntactic complexity measurement.

### L2 syntactic complexity analyzer

Syntactic complexity is recognized as a multidimensional construct rather than a unitary linguistic feature [67]. Consequently, no single index can adequately capture its overall complexity. Accordingly, the present study employed the 14 indices generated by the L2 Syntactic Complexity Analyzer (L2SCA) to provide a multidimensional description of the syntactic characteristics of the two corpora. Although L2SCA was originally developed for the analysis of L2 writing, it has been widely adopted in studies of translated English to quantify syntactic complexity [2,6,35,38–40,44].

In the present study, L2SCA is used to analyze the syntactic complexity of TTAE and NAE. It is an automatic syntactic complexity analyzer originally designed for second language writing, with fourteen measures that have been explored or proposed in studies of second language development [29]. Table 3 displays these fourteen measures, their codes, definitions, and the dimensions they belong to. There are altogether five types of measures, including length of production unit, sentence complexity, subordination, coordination, and particular structures. “Code” and “definition” are built into the analyzer. The “Code” refers to the abbreviations of measures, and the “definition” refers to the calculation method of measures. Detailed information about the definitions and operations can be found in Lu’s research [29], which explains that for all measures, a higher score suggests a greater degree of syntactic complexity.

**Table 3:** Syntactic complexity measures based on Lu (2010: 479) [29].

Measure	Code	Definition
Type 1: Length of production unit		
Mean length of clause	MLC	# of words / # of clauses
Mean length of sentence	MLS	# of words / # of sentences
Mean length of T-unit	MLT	# of words / # of T-units
Type 2: Sentence complexity		
Sentence complexity ratio	C/S	# of clauses / # of sentences
Type 3: Subordination		
T-unit complexity ratio	C/T	# of clauses / # of T-units
Complex T-unit ratio	CT/T	# of complex T-units / # of T-units
Dependent clause ratio	DC/C	# of dependent clauses / # of clauses
Dependent clause per T-unit	DC/T	# of dependent clauses / # of T-units
Type 4: Coordination		
Coordinate phrases per clause	CP/C	# of coordinate phrases / # of clauses
Coordinate phrases per T-unit	CP/T	# of coordinate phrases / # of T-units
Sentence coordination ratio	T/S	# of T-units / # of sentences
Type 5: Particular structures		
Complex nominals per clause	CN/C	# of complex nominals / # of clauses
Complex nominals per T-unit	CN/T	# of complex nominals / # of T-units
Verb phrases per T-unit	VP/T	# of verb phrases / # of T-units

## Section 4: Results

In this section, through a comparative analysis of syntactic complexity in TTAE and NAE, the overall differences of the two corpora are identified, and the specific syntactic characteristics of TTAE are further explored with NAE as the benchmark.

### Differences in syntactic complexity between TTAE and NAE

The statistics presented in Table 4 provide an overview of the syntactic complexity that distinguishes TTAE from NAE. The mean values and standard deviations of the 14 syntactic complexity measures of the two corpora show differences across various dimensions. This divergence is not characterized by a uniform defect on the part of trainees, but rather a distinct reconfiguration of lexical and syntactic resources. As shown in Table 3, the syntactic complexity of TTAE is relatively higher in terms of the length of the production units (MLS, MLC, and MLT), coordination (CP/C, CP/T), and particular structures of complex nominals (CN/C, CN/T). In contrast, NAEs are more complex in the level of sentence complexity (C/S) and subordination (C/T, CT/T, DC/C, DC/T). The result is broadly in line with the findings reported by Tan Hua, which indicate that the syntactic complexity of Chinese expert-translated academic texts is higher than native English academic writings in measures of MLC, CP/C, CP/T, T/S, CN/C, and CN/T [47].

**Table 4:** Mean values for 14 Syntactic Complexity Measures.

Dimensions of Measures	Code	TTAE	(SD)	NAE	(SD)
Length of production unit	MLC	14.33*	2.36	12.59	2.36
	MLS	26.38*	5.48	24.72	4.56
	MLT	22.54*	3.71	21.34	3.39
Sentence complexity	C/S	1.85	0.27	2.00*	0.41
Subordination	C/T	1.58	0.17	1.72*	0.27
	CT/T	0.40	0.09	0.47*	0.12
	DC/C	0.34	0.06	0.39*	0.08
	DC/T	0.54	0.14	0.69*	0.24
Coordination	CP/C	0.52*	0.19	0.40	0.17
	CP/T	0.82*	0.31	0.66	0.23
	T/S	1.16	0.08	1.16	0.09
Particular structures	CN/C	1.95*	0.46	1.62	0.41
	CN/T	3.06*	0.73	2.74	0.65
	VP/T	2.11	0.27	2.24*	0.38

Note: * indicates higher syntactic complexity.

To ensure methodological rigor and analytical robustness of subsequent analyses, a complementary suite of statistical tests was employed. The Kolmogorov-Smirnov test is used to examine the normality of the data. Statistics show that all indicators, except for CP/T, follow a normal distribution. Based on the Kolmogorov-Smirnov test, for variables that are normally distributed and meet the assumption of homogeneity of variance, independent samples t-tests are employed. For those that either do not meet the assumption of homogeneity of variance or are not normally distributed, the Mann-Whitney U test is used. The results are displayed in Table 5 and 6. Independent-samples t tests revealed significant differences for MLC, DC/C, DC/T, C/T, CT/T, CP/C, CN/T, and CN/C, whereas no significant differences were found for MLS, MLT, C/S, VP/T, and T/S. As CP/T did not satisfy the assumption of normality, a Mann–Whitney U test was performed. The analysis also revealed a significant between-group difference in CP/T.

**Table 5:** Independent Samples t‑test results for all indicators.

	Independent Samples F	*df*	*t*	*p*	Cohen’s d	95% CI of d
MLC	.011	74	3.202	.002*	0.735	[0.270, 1.200]
MLS	.574	74	1.431	.157	0.328	[-0.124, 0.781]
MLT	.540	74	1.474	.145	0.338	[-0.115, 0.791]
C/S	2.743	74	1.974	.052	−0.453	[-0.908, 0.003]
C/T	5.861	74	2.700	.009*	−0.619	[-1.080, -0.159]
CT/T	3.380	74	2.853	.006*	−0.655	[-1.117, -0.193]
DC/C	3.453	74	3.246	.002*	−0.745	[-1.210, -0.279]
DC/T	6.029	74	3.267	.002*	−0.750	[-1.215, -0.284]
CP/C	1.155	74	3.089	.003*	0.709	[0.245, 1.173]
T/S	.101	74	.396	.693	0.091	[-0.359, 0.541]
CN/C	.280	74	3.302	.001*	0.758	[0.292, 1.223]
CN/T	.984	74	2.006	.049*	0.460	[0.004, 0.916]
VP/T	3.592	74	1.728	.088	−0.397	[-0.851, 0.058]

**Note.** *df* = degrees of freedom*; p* = two‑tailed significance level (bold values indicate p < .05); Cohen’s d = standardized mean difference; 95% CI = confidence interval for Cohen’s d.

* indicates statistically significant differences (p < 0.05).

**Table 6:** Result of Mann-Whitney U Tests.

	Z for Mann-Whitney U Tests	*df*	*p*	Cohen’s d	95% CI of d
CP/T	2.608	74	.009*	0.30	[0.08, 0.49]

**Note.** *df* = degrees of freedom; *p* = two‑tailed significance level (bold values indicate p < .05); Cohen’s d = standardized mean difference; 95% CI = confidence interval for Cohen’s d.

* indicates statistically significant differences (p < 0.05).

Among these statistically significant measures, the syntactic complexity of native writers is higher in subordination, including C/T, CT/T, DC/C, and DC/T, indicating a higher level of clausal complexity. In comparison, the syntactic complexity of TTAE is higher in particular structures of CN/T and CN/C, coordination of CP/T and CP/C, and he length of production of MLC, exhibiting greater reliance on longer clauses, coordination structures and complex nominals. Thus, the syntactic complexity of academic discourse produced by native authors and that of translated texts by trainee translators exhibits significant differences across various types of syntactic complexity measures. Although Yang compared MTI students with expert translators rather than native academic writers [68], both studies reveal convergent patterns in several dimensions of syntactic complexity. Specifically, the MTI trainee students exhibit higher values in CN/T, CN/C, CP/T, CP/C, and MLC, although the two studies differ in T/S. Despite the different comparison groups, these similarities suggest that greater reliance on longer clauses, complex nominals, and coordinated structures may represent recurring syntactic characteristics of Chinese trainee translators’ academic English.

In summary, the overall syntactic complexity of the two corpora shows different features. NAE tends to favor vertical complexity through subordination, while TTAE leans toward horizontal complexity through phrasal expansion and coordination. The following sections will categorize and examine these five differences in detail.

### Specific features of TTAE compared with NAE

Based on the descriptive statistics, significance tests and effect size analysis presented in previous section, this section focuses on the distinctive syntactic complexity profile of TTAET by using NAET as a benchmark.

#### Length of production unit.

**Fig 1:**
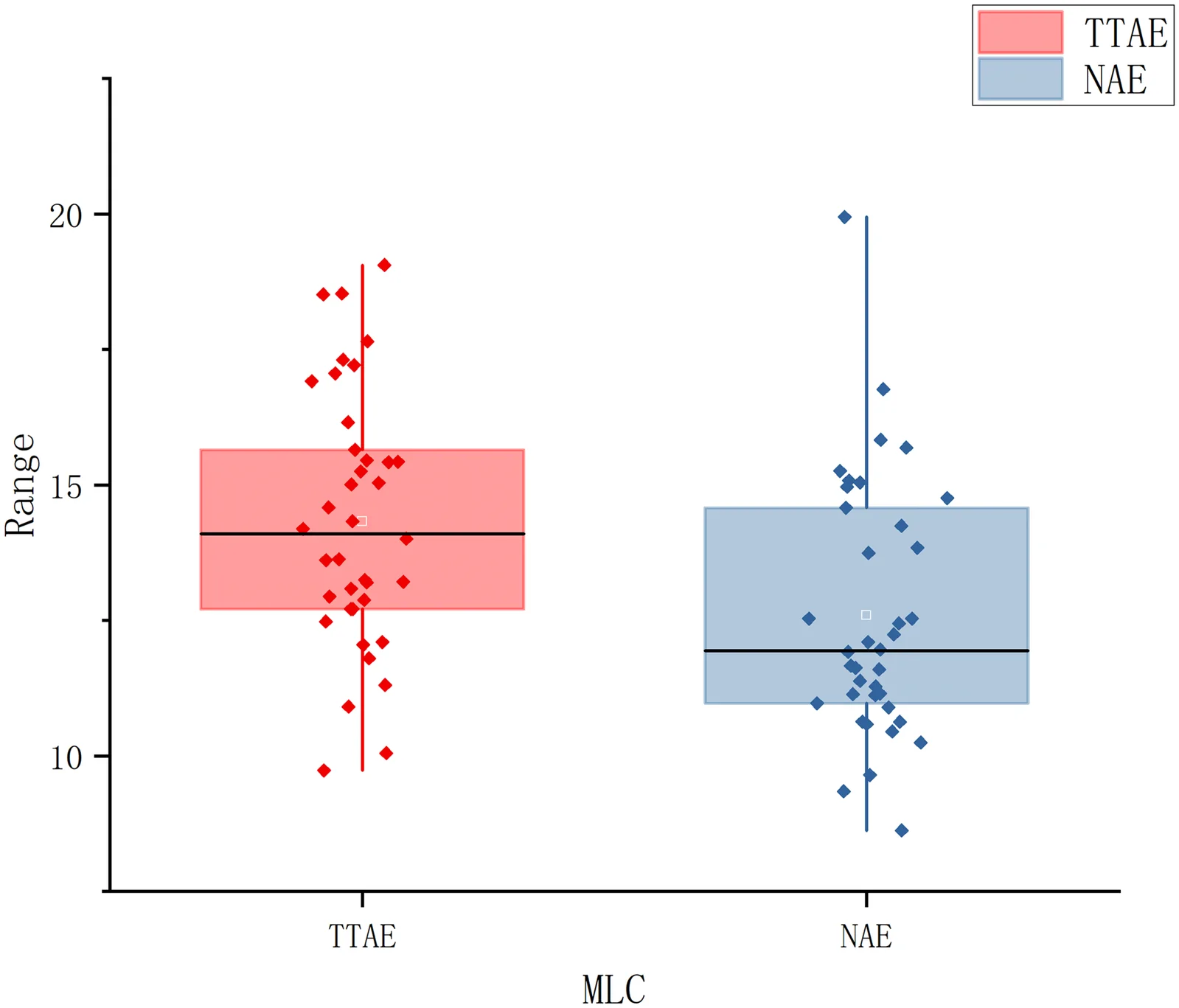
Length of Production Unit (MLC).

For length per production unit, only the MLC (Mean Length of Clause) measure shows a statistically significant difference between TTAE and NAE. As shown in Fig 1, the mean length of clauses in academic monographs written by native authors is significantly shorter than that in translated texts by trainee translators. Given that there is no notable difference between the academic English of the two groups in terms of mean length of T-unit and mean length of sentence, trainee translators tend to construct longer clauses than native academic writers, highlighting clause length as a distinctive feature of TTAE.

The measure of MLC consistently appears to be higher in translated English than in comparable native English, regardless of the comparison being made. This tendency has been observed not only in comparisons between TTAE and NAE in the present research, but also between expert translated academic English and native academic English [42,47], even between trainee and expert translators [69], with trainee translators typically producing the longer clauses. Such consistency suggests that elevated MLC appears to represent a structural tendency inherent in translated discourse.

#### Subordination structures.

**Fig 2:**
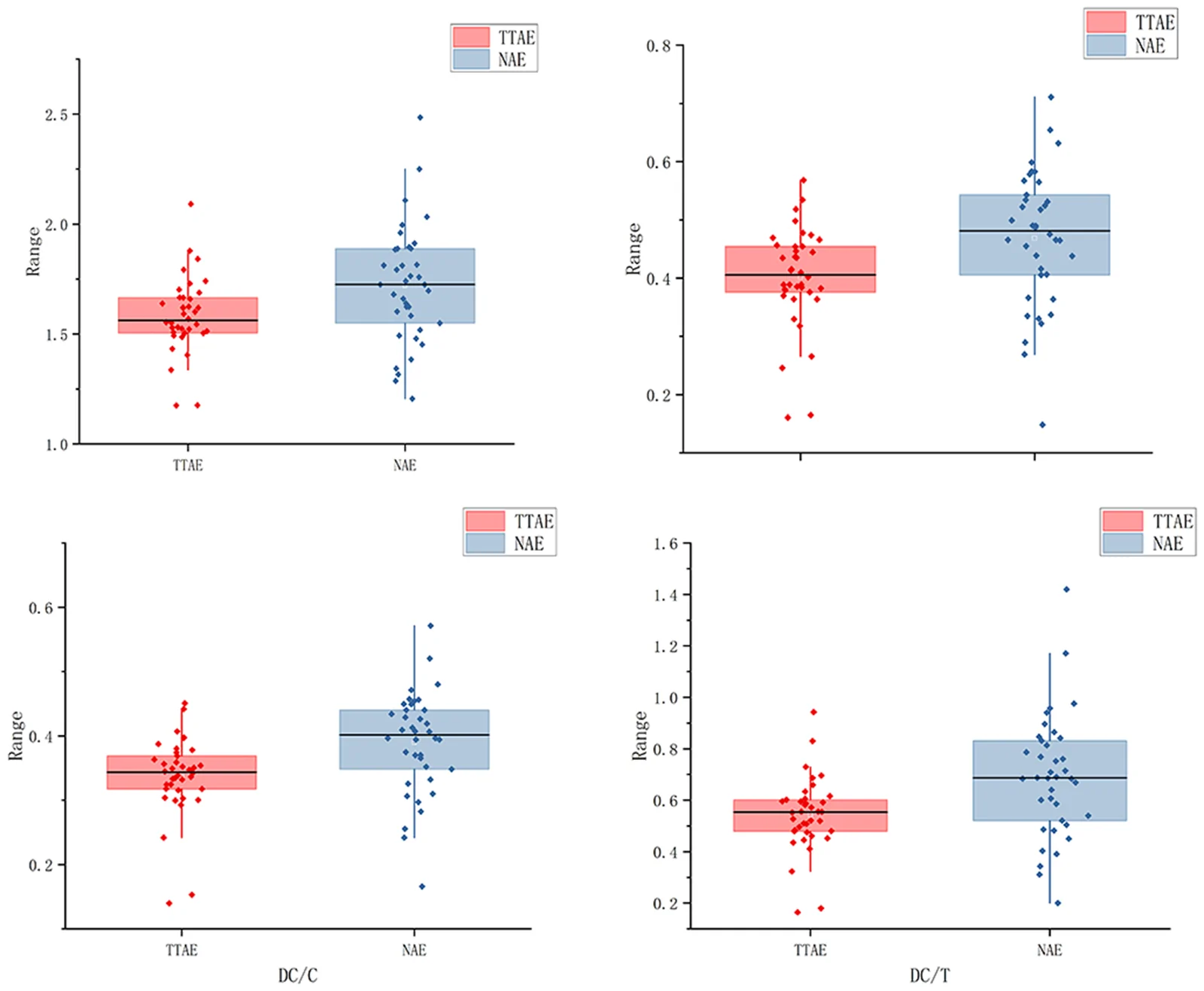
Data of Subordination (C/T, CT/T, DC/C, DC/T).

According to the boxplot analysis of Fig 2, all four measures of subordination in TTAE, C/T, CT/T, DC/C, and DC/T are lower degree of dispersion than those in NAE. Different from NAE, in terms of subordinate structures, the syntactic complexity of TTAE demonstrates a significantly lower level than that of NAE. Trainee translators tend to use fewer subordinate clauses and produce simpler T-units when rendering academic texts, while native authors are more flexible in using complex T-units and complex clauses. Rather than attributing this to the deficiency of trainee translators’ performance, it may indicate a developmental stage or a strategic preference.

#### Coordination structures.

**Fig 3:**
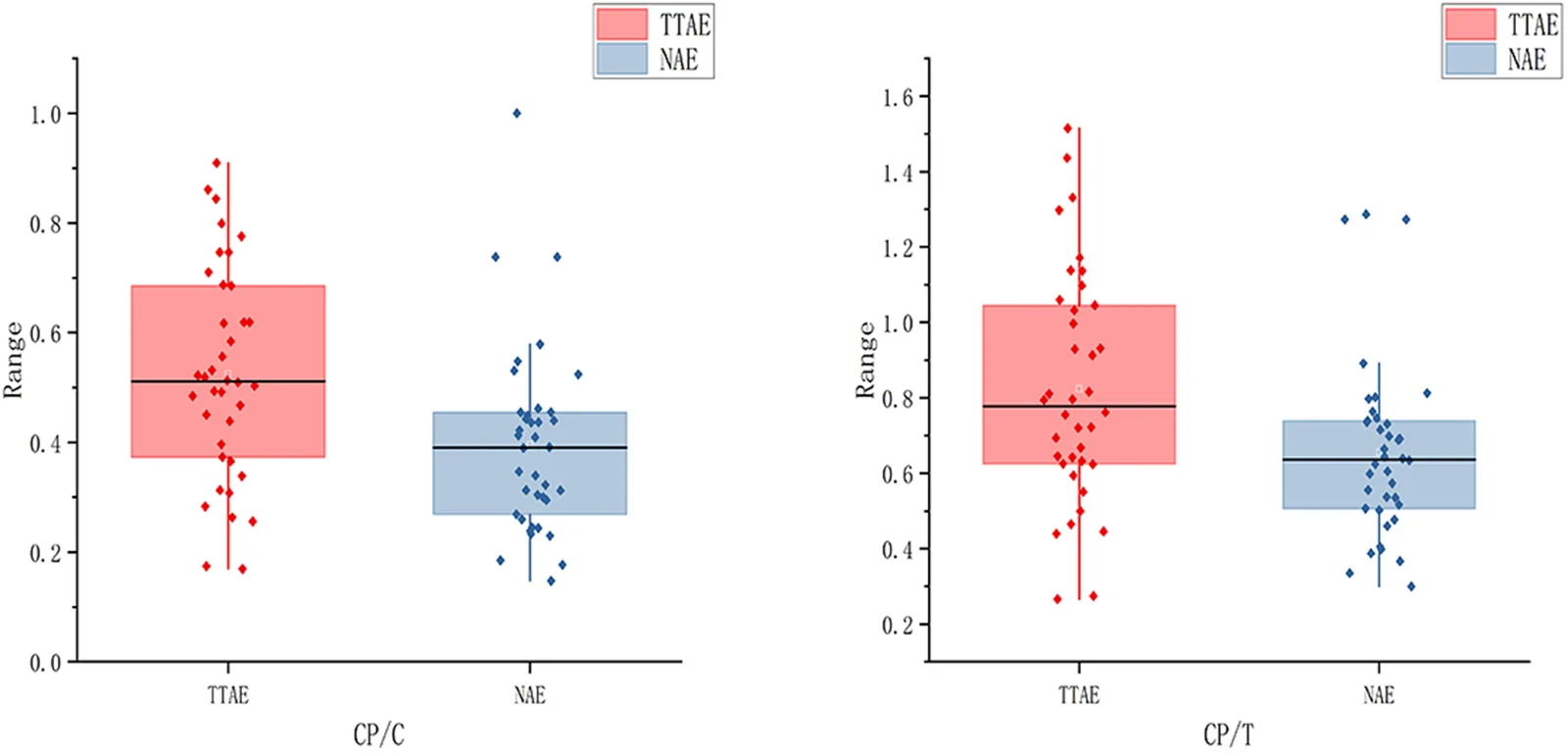
Data of Coordination(CP/C, CP/T).

As shown in Fig 3, the coordination structures of TTAE are significantly higher than those of NAE and demonstrate a wider range of dispersion. The results suggest that NAE generally use fewer coordinate phrases and adopt them more selectively in academic writing. In contrast, trainee translators tend to rely more frequently on coordinate phrases in their translations, indicating a heavier dependence on coordination as a syntactic resources. The increased use of coordinate phrases by trainee translators may reflect a preference for syntactically simpler and more linear sentence construction. Coordination requires less grammatical embedding than subordination, making it cognitively less demanding for trainee translators who are still developing their syntactic awareness in the target language. This result is in accordance with Chen et al.’s findings that expert translation texts, as “constrained languages”, tend to use longer yet simpler sentences and rely more on coordination than complex subordination, forming a stark contrast with the writing style of native speakers [6].

#### Particular structures reflecting syntactic complexity.

**Fig 4:**
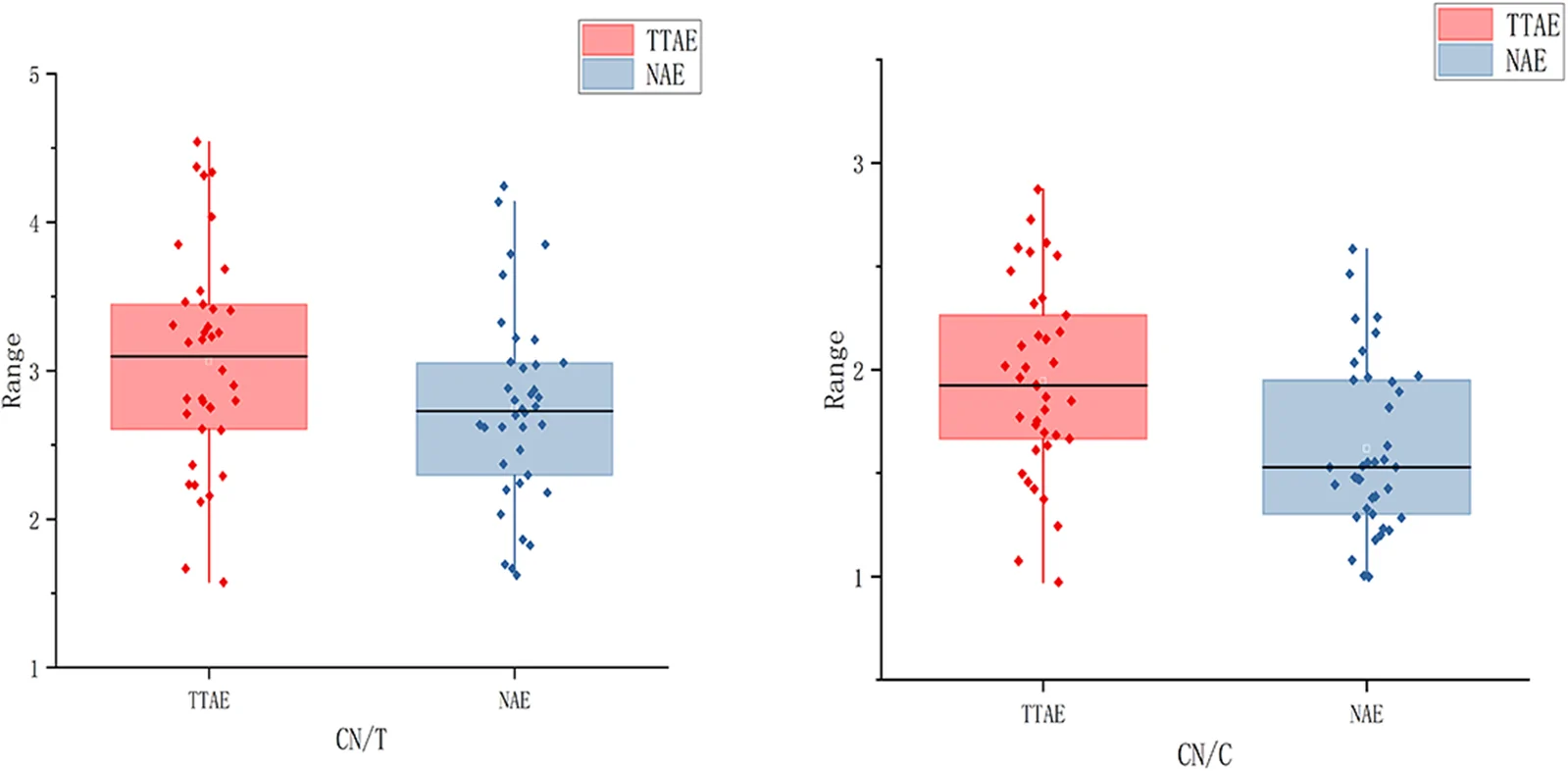
Particular Structures Influencing Syntactic Complexity (CN/C, CN/T).

According to Fig 4, CN/C and CN/T measures show statistically significant differences. The syntactic complexity of TTAE is higher than that of NAE in terms of the CN/C and CN/T indicators. This suggests that trainee translators tend to favor complex noun phrases when producing academic translations. At the same time, although native authors tend to adopt more verb phrases (the mean value for VP/T of TTAE is 2.11, while NAE is 2.24), the difference is not that significant. These findings suggest that, similar to L1 Chinese writers who tend to use complex nominals, L1 Chinese trainee translators also exhibit a preference for complex nominals in academic writing [16]. Sari’s research reveals that trainee translators’ errors include incorrect vocabulary, inappropriate use of prepositions, and incomplete sentences, which may partly lead to differences in phrase complexity between the two groups [70].

#### Sentence complexity.

**Fig 5:**
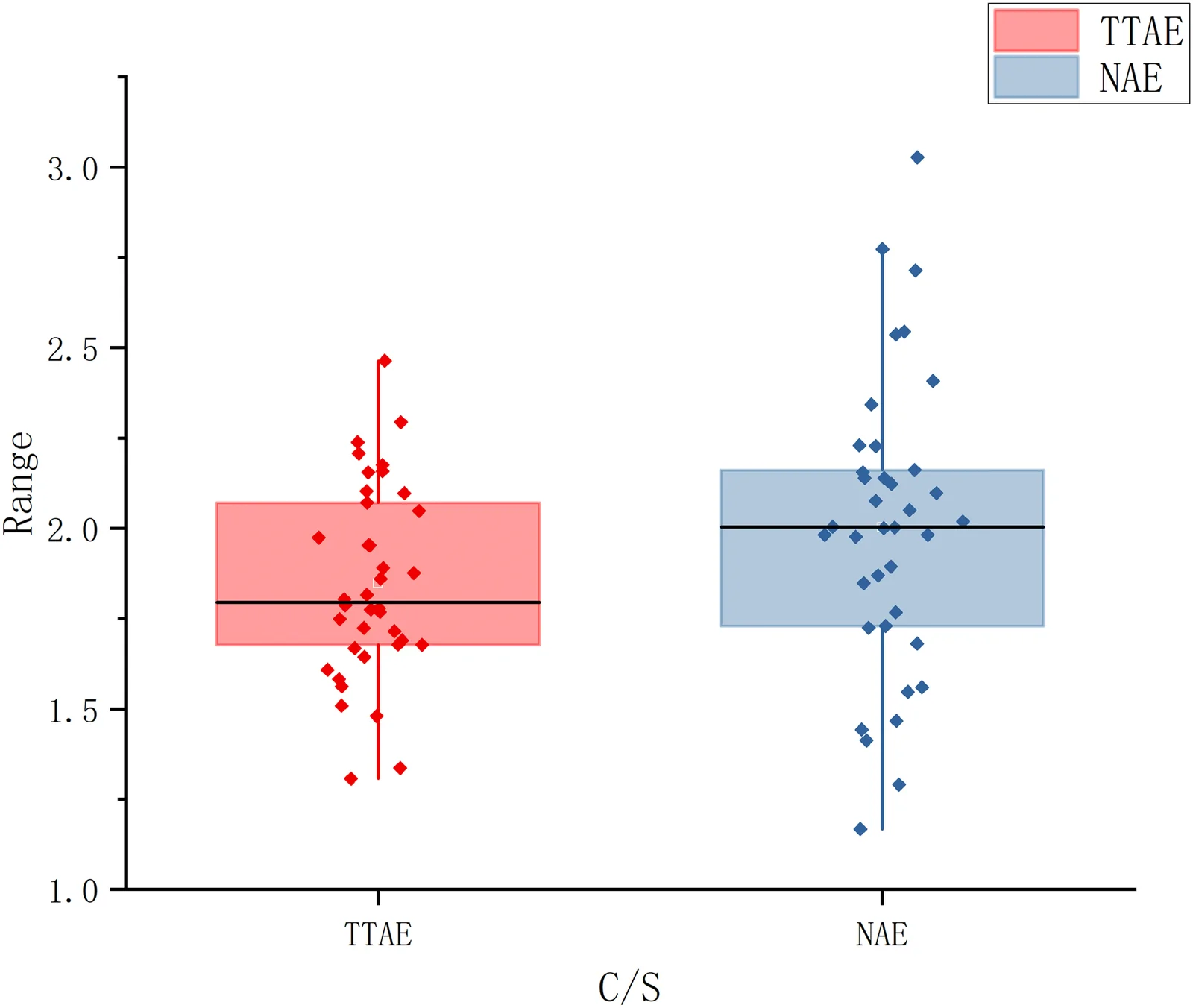
Results of Sentence complexity.

Fig 5 shows that the boxplot distributions in sentence complexity of the two types of texts. The TTAE demonstrates lower sentence complexity than that of the NAE, though the difference is not statistically significant. Concerning the dispersion degree of sentence complexity, the TTAE is more restricted in range than that of the NAE. Therefore, compared with native authors, trainee translators tend to use simpler sentence patterns..This tendency partly reflect the developmental characteristics of trainee translators as L2 language users, as L2 academic writing has been reported as a late developed competence [71].

## Section 5: Discussion

The present article has compared the syntactic complexities of English academic texts translated by trainee translators and written by native writers. Our results show that trainee translators and native authors vary their choices in employing complex syntactic structures. The comparison reveals significant differences in nine measures out of fourteen, namely, C/T, CT/T, DC/C, DC/T, CN/T, CN/C, CP/T, CP/C, and MLC. Compared to native speaking authors, the syntactic complexity of TTAE is higher in CN/T, CN/C, CP/T, CP/C, and MLC, while lower in C/T, CT/T, DC/C, and DC/T. There are no significant differences in the other five measures, that is, MLS, MLT, C/S, T/S, and VP/T.

Compared with NAE, TTAE demonstrates a distinctive profile in this study. Specifically, TTAET tends to construct longer clauses that package information through phrasal elaboration of complex nominals and coordinated structures, rather than through subordinate structures. These features will be discussed separately with reference to Excerpts (1–2), although they should be understood as interrelated dimensions of the multidimensional construct of syntactic complexity [67].

Excerpt (1): The instruction from Wilson shall be our criterion for researchers on ethical education, namely the education in social ethics and its implementation. Realistic issues and existence circumstances must be replied with wisdom, a definite target therefore can be seen in our view. (TTAE-28)

Source text: 威尔逊的告诫, 应该成为我们开展伦理教育的准则, 即探讨社会伦理教育及其实施, 必须末年至地面对现实问题和存在境遇, 以此才能做到有的放矢

Excerpt (2): Results from this kind of immersion program, such as those initiated in Canada but which now also exist elsewhere, showed that learners could indeed become quite fluent in an L2 through exposure without explicit instruction, and that they developed excellent receptive skills. (NAE-31)

### Longer and self-contained clauses

A key syntactic feature of TTAE is the reliance on longer and self-contained clauses rather than flexible clause integration, demonstrated through the higher mean length of clauses (MLC). In excerpt 1, trainee translators tends to accumulate successive phrasal modifiers within a single clause, rather than structuring the information into embedded structures. Expressions such as *from Wilson*, *for researchers on ethical education*, and *namely the education in social ethics and its implementation* are all attached to the same clause,substantially increasing the internal length of the clause.

Such reliance may reflect a more linear and literal translation strategy, possibly influenced by the syntactic structure of the source language (L1 Chinese), which often prefers parataxis and extended clauses over hypotaxis. The extended clause length in trainee translations may also be linked to the cognitive constraints of novice translators, who might focus more on transferring content accurately at the clause level rather than restructuring information syntactically to suit the conventions of academic English [42]. This results in denser clause constructions that, while grammatically correct, may reduce textual readability and stylistic elegance.

### Coordination over subordination

The tendency of coordinate structures over subordinate structures constitutes a striking feature of TTAE. The contrast between the above two Excerpts shows different syntactic organizations adopted by TTAE and NAE. The TTAE relies more on a sequence of coordinate (e.g., *The education in social ethics and its implementation, Realistic issues and existence circumstances*), whereas the NAE organize information through hierarchical subordinate structures. The preference of coordination and reduction of subordination in translated texts has also been reported in previous studies. However, existing evidence has primarily been drawn from comparisons between trainee and expert translators [42,68] or between expert-translated texts and native English texts [46]. The present findings extend this line of evidence by showing that the same tendency is also evident in comparisons between TTAE and NAE.

Compared with subordination, coordinations are at earlier stage of language development [72]. Trainee translators use less or uniform subordinate structures might mean that their English represents a developing interlanguage system.Wolfe-Quintero et al. also propose that syntactic complexity in L2 expands from coordination to subordination as learners gain proficiency [8] This partly aligns with findings by Kyle and Crossley that higher syntactic diversity is often associated with greater writing proficiency and rhetorical sophistication [73].

### Complex nominal constructions

TTAE demonstrates a preference of complex nominal constructions.

In Excerpt I, TTAE repeats noun phrases with multiple modifiers. Rather than condensing information into compact nominals, these noun phrases tend to preserve the sequence of the source text by adding prepositional and coordinated modifiers.

This may be attributed to more than one factor. Source text effect and cross-linguistic influence are imprtant ones. According to Wu et al. and Shen et al., L1 Chinese academic writing frequently favors nominalization and abstract noun-heavy structures [5], [16]. Ruan also poits out that Chinese authors tend to use more complex noun phrases to package information in English abstracts [74]. Moreover, the favor of complex nominals in TTAE may reflects the effect of style standardization [75]. Trainee translators try to adhere the formal style of academic discourse by frequently using nominalizations and nominal groups.

The findings have significant implications for translator teaching. Translation training should lay emphasis on syntactic variations in teaching input, including verbal groups, subordinate clauses, and complex sentences. Exercises on imitation of NAE discourses will help trainees expand their grammatical repertoire and enhance rhetorical sensitivity. Cultivating the awareness of the differences in syntactic patterns between the source language and target language will effectively avoid negative transfers. Our research will enriches the syntactic complexity studies by expanding to new subjects of trainee translators, a group that merits more scholarly attention.

## Section 6: Conclusion

This study employed the syntactic complexity measurement tool L2SCA to investigate the differences in syntactic complexity between TTAE and NAE, thereby identifying the linguistic features of trainees’ academic translations and providing insights for translator training. The results show significant differences between the two in various syntactic measures. Of the nine indicators with statistically significant differences, the syntactic complexity of NAE is higher in C/T, CT/T, DC/C, and DC/T, while the syntactic complexity of TTAE is higher in CN/T, CN/C, CP/T, CP/C, and MLC. Compared with native authors who tend to use shorter clauses, more subordinate clauses, and more complex T-units, particularly embedded subordination, trainee translators’ academic English prioritizes accuracy and lexical density over syntactic variety and rhetorical strategies, exemplified by longer clauses, coordinated structures, complex noun phrases, and relatively reduced syntactic dispersion. The differences may stem from the influences of source-language effect, L2 development and the translation strategies employed by trainees translators. However, since the present study did not systematically examine the causes of the distinctive features of TTAE, the extent to which the observed patterns reflect these factors cannot be determined..

Through analyzing the syntactic complexity differences between NAE and TTAE, this research provides insights for the translation pedagogy. It further suggests that translation training should encourage learners to enhance syntactic richness and flexibility by fostering greater syntactic variety, particularly in the use of subordination and complex T-units, while avoiding excessive reliance on a limited range of syntactic structures.

However, several limitations should be acknowledged. Firstly, the sample was limited to the MTI students, which may affect the generalizability of the findings. Future studies could benefit from expanding the dataset to include a more diverse group of translators and levels of translation experience. Secondly, the study focused exclusively on academic monographs, thus restricting the applicability of the results to other genres of translation, such as journal articles, reports, or oral interpreting. Genre-specific syntactic patterns may vary considerably, and further research is needed to examine whether similar trends persist across other academic and professional contexts. Thirdly, although the Chinese source texts and the native English academic texts were matched in genre, size and disciplinary distribution, they did not be empirically matched in terms of style, rhetorical structure and readability. The present study did not explicitly quantify and control the influence of the Chinese source texts on the syntactic complexity of the translations. Future research could incorporate source-text analyses to better distinguish translation-induced features from source-language influence. Finally, the present study concentrate on the measures showing statistically significant differences. Given the multidimentional nature of syntactic complexity, the remaining measures may also offer valuable insights into syntactic features of TTAE. However, owing to space limitation, they were not examined in detail and merit further investigation in future research.

### Declaration of generative AI and AI-assisted technologies in the writing process

During the preparation of this paper, the authors used ChatGPT (GPT5.5) solely to improve the language and readability of the manuscript. The authors reviewed and edited the content as needed and take full responsibility for the content of the publication.

## Supporting information

S1 Data Dataset. (DOCX)
