# Supplementary material for: A comparative analysis of syntactic complexity in English academic texts by Chinese MTI trainee translators and native academic writers
Source: PLoS One. 2026 Sep 8;21(9):e0357458. doi: 10.1371/journal.pone.0357458 (PMC13552736; doi:10.1371/journal.pone.0357458)
Supplement: Supplementary file 1 [file pone.0357458.s001.docx]

|  | W | S | VP | C | T | DC | CT | CP | CN | MLS | MLT | MLC | C/S | VP/T | C/T | DC/C | DC/T | T/S | CT/T | CP/T | CP/C | CN/T | CN/C |
| --- | --- | --- | --- | --- | --- | --- | --- | --- | --- | --- | --- | --- | --- | --- | --- | --- | --- | --- | --- | --- | --- | --- | --- |
| trainee1 |  | 7527 | 354 | 701 | 553 | 386 | 168 | 150 | 249 | 1085 21.2627 | 19.5 | 13.6112 | 1.5621 | 1.8161 | 1.4326 | 0.3038 | 0.4352 |  | 1.0904 0.3886 | 0.6451 | 0.4503 | 2.8109 | 1.962 |
| trainee2 |  | 12117 | 435 | 953 | 750 | 492 | 251 | 179 | 560 | 1740 27.8552 | 24.628 | 16.156 | 1.7241 | 1.937 | 1.5244 | 0.3347 | 0.5102 |  | 1.131 0.3638 | 1.1382 | 0.7467 | 3.5366 | 2.32 |
| trainee3 |  | 10747 | 482 | 794 | 630 | 536 | 88 | 86 | 335 | 1267 22.2967 | 20.0504 | 17.0587 | 1.3071 | 1.4813 | 1.1754 | 0.1397 | 0.1642 |  | 1.112 0.1604 | 0.625 | 0.5317 | 2.3638 | 2.0111 |
| trainee4 |  | 10121 | 374 | 901 | 766 | 440 | 271 | 205 | 196 | 1276 27.0615 | 23.0023 | 13.2128 | 2.0481 | 2.0477 | 1.7409 | 0.3538 | 0.6159 |  | 1.1765 0.4659 | 0.4455 | 0.2559 | 2.9 | 1.6658 |
| trainee5 |  | 5413 | 199 | 398 | 320 | 206 | 124 | 94 | 167 | 793 27.201 | 26.2767 | 16.9156 | 1.608 | 1.932 | 1.5534 | 0.3875 | 0.6019 |  | 1.0352 0.4563 | 0.8107 | 0.5219 | 3.8495 | 2.4781 |
| trainee6 |  | 16804 | 578 | 1763 | 1424 | 681 | 642 | 387 | 375 | 2326 29.0727 | 24.6755 | 11.8006 | 2.4637 | 2.5888 | 2.091 | 0.4508 | 0.9427 |  | 1.1782 0.5683 | 0.5507 | 0.2633 | 3.4156 | 1.6334 |
| trainee7 |  | 12770 | 380 | 1018 | 742 | 494 | 251 | 215 | 510 | 2132 33.6053 | 25.8502 | 17.2102 | 1.9526 | 2.0607 | 1.502 | 0.3383 | 0.5081 |  | 1.3 0.4352 | 1.0324 | 0.6873 | 4.3158 | 2.8733 |
| trainee8 |  | 7350 | 251 | 611 | 397 | 297 | 96 | 73 | 206 | 1028 29.2829 | 24.7475 | 18.5139 | 1.5817 | 2.0572 | 1.3367 | 0.2418 | 0.3232 |  | 1.1833 0.2458 | 0.6936 | 0.5189 | 3.4613 | 2.5894 |
| trainee9 |  | 6400 | 281 | 612 | 497 | 306 | 175 | 119 | 153 | 843 22.7758 | 20.915 | 12.8773 | 1.7687 | 2 | 1.6242 | 0.3521 | 0.5719 |  | 1.089 0.3889 | 0.5 | 0.3078 | 2.7549 | 1.6962 |
| trainee10 |  | 13339 | 524 | 989 | 700 | 595 | 107 | 98 | 543 | 1787 25.4561 | 22.4185 | 19.0557 | 1.3359 | 1.6622 | 1.1765 | 0.1529 | 0.1798 |  | 1.1355 0.1647 | 0.9126 | 0.7757 | 3.0034 | 2.5529 |
| trainee11 |  | 9959 | 529 | 961 | 798 | 522 | 275 | 193 | 139 | 1414 18.8261 | 19.0785 | 12.4799 | 1.5085 | 1.841 | 1.5287 | 0.3446 | 0.5268 |  | 0.9868 0.3697 | 0.2663 | 0.1742 | 2.7088 | 1.7719 |
| trainee12 |  | 6441 | 230 | 577 | 454 | 277 | 165 | 130 | 220 | 916 28.0043 | 23.2527 | 14.1872 | 1.9739 | 2.083 | 1.639 | 0.3634 | 0.5957 |  | 1.2043 0.4693 | 0.7942 | 0.4846 | 3.3069 | 2.0176 |
| trainee13 |  | 7971 | 309 | 795 | 530 | 331 | 184 | 133 | 270 | 1078 25.7961 | 24.0816 | 15.0396 | 1.7152 | 2.4018 | 1.6012 | 0.3472 | 0.5559 |  | 1.0712 0.4018 | 0.8157 | 0.5094 | 3.2568 | 2.034 |
| trainee14 |  | 9183 | 354 | 1189 | 812 | 441 | 307 | 209 | 275 | 1010 25.9407 | 20.8231 | 11.3091 | 2.2938 | 2.6961 | 1.8413 | 0.3781 | 0.6961 |  | 1.2458 0.4739 | 0.6236 | 0.3387 | 2.2902 | 1.2438 |
| trainee15 |  | 12185 | 536 | 1246 | 894 | 584 | 290 | 227 | 441 | 1302 22.7332 | 20.8647 | 13.6298 | 1.6679 | 2.1336 | 1.5308 | 0.3244 | 0.4966 |  | 1.0896 0.3887 | 0.7551 | 0.4933 | 2.2295 | 1.4564 |
| trainee16 |  | 6277 | 293 | 682 | 521 | 332 | 173 | 136 | 389 | 716 21.4232 | 18.9066 | 12.048 | 1.7782 | 2.0542 | 1.5693 | 0.3321 | 0.5211 |  | 1.1331 0.4096 | 1.1717 | 0.7466 | 2.1566 | 1.3743 |
| trainee17 |  | 7907 | 237 | 685 | 448 | 270 | 178 | 129 | 307 | 1171 33.3629 | 29.2852 | 17.6496 | 1.8903 | 2.537 | 1.6593 | 0.3973 | 0.6593 |  | 1.1392 0.4778 | 1.137 | 0.6853 | 4.337 | 2.6138 |
| trainee18 |  | 7170 | 215 | 641 | 464 | 247 | 205 | 132 | 271 | 997 33.3488 | 29.0283 | 15.4526 | 2.1581 | 2.5951 | 1.8785 | 0.4418 | 0.83 |  | 1.1488 0.5344 | 1.0972 | 0.5841 | 4.0364 | 2.1487 |
| trainee19 |  | 8205 | 404 | 838 | 678 | 439 | 228 | 165 | 317 | 1141 20.3094 | 18.6902 | 12.1018 | 1.6782 | 1.9089 | 1.5444 | 0.3363 | 0.5194 |  | 1.0866 0.3759 | 0.7221 | 0.4676 | 2.5991 | 1.6829 |
| trainee20 |  | 19779 | 1103 | 2162 | 1813 | 1220 | 543 | 402 | 567 | 2581 17.932 | 16.2123 | 10.9095 | 1.6437 | 1.7721 | 1.4861 | 0.2995 | 0.4451 |  | 1.1061 0.3295 | 0.4648 | 0.3127 | 2.1156 | 1.4236 |
| trainee21 |  | 15902 | 595 | 1581 | 1251 | 772 | 449 | 337 | 496 | 2170 26.7261 | 20.5984 | 12.7114 | 2.1025 | 2.0479 | 1.6205 | 0.3589 | 0.5816 |  | 1.2975 0.4365 | 0.6425 | 0.3965 | 2.8109 | 1.7346 |
| trainee22 |  | 16025 | 558 | 1632 | 1214 | 702 | 482 | 319 | 444 | 2267 28.7186 | 22.8276 | 13.2002 | 2.1756 | 2.3248 | 1.7293 | 0.397 | 0.6866 |  | 1.2581 0.4544 | 0.6325 | 0.3657 | 3.2293 | 1.8674 |
| trainee23 |  | 5519 | 325 | 742 | 549 | 339 | 188 | 90 | 93 | 534 16.9815 | 16.2802 | 10.0528 | 1.6892 | 2.1888 | 1.6195 | 0.3424 | 0.5546 |  | 1.0431 0.2655 | 0.2743 | 0.1694 | 1.5752 | 0.9727 |
| trainee24 |  | 6485 | 276 | 650 | 463 | 306 | 147 | 117 | 233 | 856 23.4964 | 21.1928 | 14.0065 | 1.6775 | 2.1242 | 1.5131 | 0.3175 | 0.4804 |  | 1.1087 0.3824 | 0.7614 | 0.5032 | 2.7974 | 1.8488 |
| trainee25 |  | 7780 | 252 | 771 | 543 | 303 | 221 | 157 | 302 | 1044 30.873 | 25.6766 | 14.3278 | 2.1548 | 2.5446 | 1.7921 | 0.407 | 0.7294 |  | 1.2024 0.5182 | 0.9967 | 0.5562 | 3.4455 | 1.9227 |
| trainee26 |  | 11651 | 443 | 1095 | 799 | 530 | 254 | 202 | 688 | 1691 26.3002 | 21.983 | 14.582 | 1.8036 | 2.066 | 1.5075 | 0.3179 | 0.4792 |  | 1.1964 0.3811 | 1.2981 | 0.8611 | 3.1906 | 2.1164 |
| trainee27 |  | 11332 | 477 | 1122 | 866 | 535 | 324 | 243 | 426 | 1395 23.7568 | 21.1813 | 13.0855 | 1.8155 | 2.0972 | 1.6187 | 0.3741 | 0.6056 |  | 1.1216 0.4542 | 0.7963 | 0.4919 | 2.6075 | 1.6109 |
| trainee28 |  | 10698 | 280 | 872 | 618 | 371 | 206 | 154 | 562 | 1685 38.2071 | 28.8356 | 17.3107 | 2.2071 | 2.3504 | 1.6658 | 0.3333 | 0.5553 |  | 1.325 0.4151 | 1.5148 | 0.9094 | 4.5418 | 2.7265 |
| trainee29 |  | 8425 | 291 | 712 | 546 | 363 | 164 | 132 | 338 | 1236 28.9519 | 23.2094 | 15.4304 | 1.8763 | 1.9614 | 1.5041 | 0.3004 | 0.4518 |  | 1.2474 0.3636 | 0.9311 | 0.619 | 3.405 | 2.2637 |
| trainee30 |  | 7023 | 226 | 642 | 468 | 281 | 178 | 140 | 374 | 902 31.0752 | 24.9929 | 15.0064 | 2.0708 | 2.2847 | 1.6655 | 0.3803 | 0.6335 |  | 1.2434 0.4982 | 1.331 | 0.7991 | 3.21 | 1.9274 |
| trainee31 |  | 9063 | 365 | 934 | 713 | 448 | 263 | 200 | 266 | 1250 24.8301 | 20.2299 | 12.7111 | 1.9534 | 2.0848 | 1.5915 | 0.3689 | 0.5871 |  | 1.2274 0.4464 | 0.5938 | 0.3731 | 2.7902 | 1.7532 |
| trainee32 |  | 12863 | 522 | 1268 | 971 | 638 | 294 | 245 | 426 | 1754 24.6418 | 20.1614 | 13.2472 | 1.8602 | 1.9875 | 1.5219 | 0.3028 | 0.4608 |  | 1.2222 0.384 | 0.6677 | 0.4387 | 2.7492 | 1.8064 |
| trainee33 |  | 8667 | 268 | 778 | 562 | 333 | 197 | 148 | 348 | 1227 32.3396 | 26.027 | 15.4217 | 2.097 | 2.3363 | 1.6877 | 0.3505 | 0.5916 |  | 1.2425 0.4444 | 1.045 | 0.6192 | 3.6847 | 2.1833 |
| trainee34 |  | 9020 | 390 | 927 | 697 | 467 | 226 | 177 | 495 | 1043 23.1282 | 19.3148 | 12.9412 | 1.7872 | 1.985 | 1.4925 | 0.3242 | 0.4839 |  | 1.1974 0.379 | 1.06 | 0.7102 | 2.2334 | 1.4964 |
| trainee35 |  | 11613 | 418 | 945 | 742 | 493 | 234 | 190 | 458 | 1606 27.7823 | 23.5558 | 15.6509 | 1.7751 | 1.9168 | 1.5051 | 0.3154 | 0.4746 |  | 1.1794 0.3854 | 0.929 | 0.6173 | 3.2576 | 2.1644 |
| trainee36 |  | 9345 | 549 | 1244 | 960 | 619 | 342 | 269 | 272 | 1032 17.0219 | 15.0969 | 9.7344 | 1.7486 | 2.0097 | 1.5509 | 0.3563 | 0.5525 |  | 1.1275 0.4346 | 0.4394 | 0.2833 | 1.6672 | 1.075 |
| trainee37 |  | 12360 | 298 | 889 | 667 | 392 | 233 | 162 | 563 | 1714 41.4765 | 31.5306 | 18.5307 | 2.2383 | 2.2679 | 1.7015 | 0.3493 | 0.5944 |  | 1.3154 0.4133 | 1.4362 | 0.8441 | 4.3724 | 2.5697 |
| trainee38 |  | 10112 | 448 | 923 | 663 | 472 | 194 | 150 | 340 | 1556 22.5714 | 21.4237 | 15.2519 | 1.4799 | 1.9555 | 1.4047 | 0.2926 | 0.411 |  | 1.0536 0.3178 | 0.7203 | 0.5128 | 3.2966 | 2.3469 |
|  | W | S | VP | C | T | DC | CT | CP | CN | MLS | MLT | MLC | C/S | VP/T | C/T | DC/C | DC/T | T/S | CT/T | CP/T | CP/C | CN/T | CN/C |
| native 1 |  | 10406 | 345 | 932 | 682 | 406 | 279 | 212 | 362 | 1680 30.1623 | 25.6305 | 15.2581 | 1.9768 | 2.2956 | 1.6798 | 0.4091 | 0.6872 |  | 1.1768 0.5222 | 0.8916 | 0.5308 | 4.1379 | 2.4633 |
| native 2 |  | 10994 | 374 | 1003 | 800 | 488 | 296 | 239 | 349 | 1571 29.3957 | 22.5287 | 13.7425 | 2.139 | 2.0553 | 1.6393 | 0.37 | 0.6066 |  | 1.3048 0.4898 | 0.7152 | 0.4363 | 3.2193 | 1.9638 |
| native 3 |  | 10149 | 437 | 830 | 641 | 463 | 181 | 149 | 280 | 1397 23.2243 | 21.9201 | 15.8331 | 1.4668 | 1.7927 | 1.3844 | 0.2824 | 0.3909 |  | 1.0595 0.3218 | 0.6048 | 0.4368 | 3.0173 | 2.1794 |
| native 4 |  | 11120 | 356 | 1001 | 739 | 408 | 332 | 238 | 327 | 1545 31.236 | 27.2549 | 15.0474 | 2.0758 | 2.4534 | 1.8113 | 0.4493 | 0.8137 |  | 1.1461 0.5833 | 0.8015 | 0.4425 | 3.7868 | 2.0907 |
| native 5 |  | 10463 | 314 | 1042 | 756 | 372 | 363 | 235 | 236 | 1432 33.3217 | 28.1263 | 13.8399 | 2.4076 | 2.8011 | 2.0323 | 0.4802 | 0.9758 |  | 1.1847 0.6317 | 0.6344 | 0.3122 | 3.8495 | 1.8942 |
| native 6 |  | 10276 | 420 | 807 | 655 | 451 | 203 | 164 | 288 | 1272 24.4667 | 22.7849 | 15.6885 | 1.5595 | 1.7894 | 1.4523 | 0.3099 | 0.4501 |  | 1.0738 0.3636 | 0.6386 | 0.4397 | 2.8204 | 1.942 |
| native 7 |  | 10495 | 629 | 1450 | 1088 | 670 | 429 | 294 | 267 | 1087 16.6852 | 15.6642 | 9.6461 | 1.7297 | 2.1642 | 1.6239 | 0.3943 | 0.6403 |  | 1.0652 0.4388 | 0.3985 | 0.2454 | 1.6224 | 0.9991 |
| native 8 |  | 10471 | 451 | 1172 | 903 | 512 | 385 | 265 | 275 | 1403 23.2173 | 20.4512 | 11.5958 | 2.0022 | 2.2891 | 1.7637 | 0.4264 | 0.752 |  | 1.1353 0.5176 | 0.5371 | 0.3045 | 2.7402 | 1.5537 |
| native 9 |  | 10191 | 316 | 946 | 681 | 380 | 292 | 203 | 281 | 1385 32.25 | 26.8184 | 14.9648 | 2.1551 | 2.4895 | 1.7921 | 0.4288 | 0.7684 |  | 1.2025 0.5342 | 0.7395 | 0.4126 | 3.6447 | 2.0338 |
| native 10 |  | 11548 | 496 | 642 | 579 | 450 | 140 | 121 | 579 | 1496 23.2823 | 25.6622 | 19.9447 | 1.1673 | 1.4267 | 1.2867 | 0.2418 | 0.3111 |  | 0.9073 0.2689 | 1.2867 | 1 | 3.3244 | 2.5838 |
| native 11 |  | 10553 | 436 | 1204 | 872 | 501 | 355 | 244 | 203 | 1353 24.2041 | 21.0639 | 12.1021 | 2 | 2.4032 | 1.7405 | 0.4071 | 0.7086 |  | 1.1491 0.487 | 0.4052 | 0.2328 | 2.7006 | 1.5516 |
| native 12 |  | 10513 | 486 | 762 | 627 | 520 | 104 | 77 | 363 | 1413 21.6317 | 20.2173 | 16.7671 | 1.2901 | 1.4654 | 1.2058 | 0.1659 | 0.2 |  | 1.07 0.1481 | 0.6981 | 0.5789 | 2.7173 | 2.2536 |
| native 13 |  | 10256 | 534 | 1147 | 921 | 617 | 300 | 226 | 239 | 1356 19.206 | 16.6224 | 11.1357 | 1.7247 | 1.859 | 1.4927 | 0.3257 | 0.4862 |  | 1.1554 0.3663 | 0.3874 | 0.2595 | 2.1977 | 1.4723 |
| native 14 |  | 11231 | 377 | 942 | 761 | 491 | 265 | 215 | 399 | 1499 29.7905 | 22.8737 | 14.7582 | 2.0186 | 1.9185 | 1.5499 | 0.3482 | 0.5397 |  | 1.3024 0.4379 | 0.8126 | 0.5243 | 3.053 | 1.9698 |
| native 15 |  | 10323 | 379 | 1143 | 888 | 453 | 406 | 262 | 346 | 1305 27.2375 | 22.7881 | 11.625 | 2.343 | 2.5232 | 1.9603 | 0.4572 | 0.8962 |  | 1.1953 0.5784 | 0.7638 | 0.3896 | 2.8808 | 1.4696 |
| native 16 |  | 10560 | 441 | 1281 | 936 | 494 | 427 | 279 | 318 | 1218 23.9456 | 21.3765 | 11.2821 | 2.1224 | 2.5931 | 1.8947 | 0.4562 | 0.8644 |  | 1.1202 0.5648 | 0.6437 | 0.3397 | 2.4656 | 1.3013 |
| native 17 |  | 11006 | 443 | 1131 | 878 | 509 | 348 | 237 | 648 | 1342 24.8442 | 21.6228 | 12.5353 | 1.9819 | 2.222 | 1.725 | 0.3964 | 0.6837 |  | 1.149 0.4656 | 1.2731 | 0.738 | 2.6365 | 1.5285 |

| native 18 | 11006 | 443 | 1131 | 878 | 509 | 348 | 237 | 648 | 1342 | 24.8442 | 21.6228 | 12.5353 | 1.9819 | 2.222 | 1.725 | 0.3964 | 0.6837 | 1.149 | 0.4656 | 1.2731 | 0.738 | 2.6365 | 1.5285 |
| --- | --- | --- | --- | --- | --- | --- | --- | --- | --- | --- | --- | --- | --- | --- | --- | --- | --- | --- | --- | --- | --- | --- | --- |
| native 19 | 10644 | 384 | 1240 | 977 | 511 | 430 | 268 | 293 | 1174 | 27.7188 | 20.8297 | 10.8946 | 2.5443 | 2.4266 | 1.9119 | 0.4401 | 0.8415 | 1.3307 | 0.5245 | 0.5734 | 0.2999 | 2.2975 | 1.2016 |
| native 20 | 10699 | 422 | 1299 | 940 | 471 | 443 | 282 | 294 | 1441 | 25.3531 | 22.7155 | 11.3819 | 2.2275 | 2.758 | 1.9958 | 0.4713 | 0.9406 | 1.1161 | 0.5987 | 0.6242 | 0.3128 | 3.0594 | 1.533 |
| native 21 | 11592 | 382 | 1262 | 969 | 513 | 354 | 208 | 236 | 1344 | 30.3455 | 22.5965 | 11.9628 | 2.5366 | 2.46 | 1.8889 | 0.3653 | 0.6901 | 1.3429 | 0.4055 | 0.46 | 0.2436 | 2.6199 | 1.387 |
| native 22 | 10552 | 491 | 1329 | 1030 | 607 | 406 | 282 | 182 | 1322 | 21.4908 | 17.3839 | 10.2447 | 2.0978 | 2.1895 | 1.6969 | 0.3942 | 0.6689 | 1.2363 | 0.4646 | 0.2998 | 0.1767 | 2.1779 | 1.2835 |
| native 23 | 10795 | 491 | 1191 | 984 | 543 | 427 | 271 | 182 | 1421 | 21.9857 | 19.8803 | 10.9705 | 2.0041 | 2.1934 | 1.8122 | 0.4339 | 0.7864 | 1.1059 | 0.4991 | 0.3352 | 0.185 | 2.6169 | 1.4441 |
| native 24 | 6354 | 393 | 887 | 608 | 411 | 214 | 167 | 196 | 749 | 16.1679 | 15.4599 | 10.4507 | 1.5471 | 2.1582 | 1.4793 | 0.352 | 0.5207 | 1.0458 | 0.4063 | 0.4769 | 0.3224 | 1.8224 | 1.2319 |
| native 25 | 10109 | 480 | 1088 | 909 | 560 | 270 | 185 | 372 | 1255 | 21.0604 | 18.0518 | 11.121 | 1.8938 | 1.9429 | 1.6232 | 0.297 | 0.4821 | 1.1667 | 0.3304 | 0.6643 | 0.4092 | 2.2411 | 1.3806 |
| native 26 | 10457 | 502 | 1412 | 1119 | 594 | 503 | 337 | 301 | 1207 | 20.8307 | 17.6044 | 9.345 | 2.2291 | 2.3771 | 1.8838 | 0.4495 | 0.8468 | 1.1833 | 0.5673 | 0.5067 | 0.269 | 2.032 | 1.0786 |
| native 27 | 10522 | 403 | 1545 | 1220 | 491 | 697 | 349 | 180 | 1492 | 26.1092 | 21.4297 | 8.6246 | 3.0273 | 3.1466 | 2.4847 | 0.5713 | 1.4196 | 1.2184 | 0.7108 | 0.3666 | 0.1475 | 3.0387 | 1.223 |
| native 28 | 11043 | 440 | 1194 | 902 | 497 | 378 | 264 | 266 | 1412 | 25.0977 | 22.2193 | 12.2428 | 2.05 | 2.4024 | 1.8149 | 0.4191 | 0.7606 | 1.1295 | 0.5312 | 0.5352 | 0.2949 | 2.841 | 1.5654 |
| native 29 | 9968 | 309 | 829 | 661 | 350 | 291 | 190 | 279 | 1485 | 32.2589 | 28.48 | 15.0802 | 2.1392 | 2.3686 | 1.8886 | 0.4402 | 0.8314 | 1.1327 | 0.5429 | 0.7971 | 0.4221 | 4.2429 | 2.2466 |
| native 30 | 11510 | 428 | 1183 | 925 | 526 | 376 | 250 | 362 | 1509 | 26.8925 | 21.8821 | 12.4432 | 2.1612 | 2.249 | 1.7586 | 0.4065 | 0.7148 | 1.229 | 0.4753 | 0.6882 | 0.3914 | 2.8688 | 1.6314 |
| native 31 | 11037 | 501 | 1206 | 926 | 578 | 347 | 263 | 321 | 1370 | 22.0299 | 19.0952 | 11.919 | 1.8483 | 2.0865 | 1.6021 | 0.3747 | 0.6003 | 1.1537 | 0.455 | 0.5554 | 0.3467 | 2.3702 | 1.4795 |
| native 32 | 13011 | 451 | 1659 | 1224 | 544 | 637 | 356 | 281 | 1745 | 28.8492 | 23.9173 | 10.6299 | 2.714 | 3.0496 | 2.25 | 0.5204 | 1.171 | 1.2062 | 0.6544 | 0.5165 | 0.2296 | 3.2077 | 1.4257 |
| native 33 | 9949 | 339 | 1154 | 940 | 446 | 427 | 260 | 224 | 1249 | 29.3481 | 22.3072 | 10.584 | 2.7729 | 2.5874 | 2.1076 | 0.4543 | 0.9574 | 1.3156 | 0.583 | 0.5022 | 0.2383 | 2.8004 | 1.3287 |
| native 34 | 13122 | 624 | 1127 | 900 | 670 | 230 | 194 | 493 | 1756 | 21.0288 | 19.5851 | 14.58 | 1.4423 | 1.6821 | 1.3433 | 0.2556 | 0.3433 | 1.0737 | 0.2896 | 0.7358 | 0.5478 | 2.6209 | 1.9511 |
| native 35 | 9941 | 500 | 1106 | 935 | 563 | 386 | 276 | 420 | 939 | 19.882 | 17.6572 | 10.6321 | 1.87 | 1.9645 | 1.6607 | 0.4128 | 0.6856 | 1.126 | 0.4902 | 0.746 | 0.4492 | 1.6679 | 1.0043 |
| native 36 | 11330 | 575 | 1261 | 1016 | 642 | 376 | 267 | 469 | 1196 | 19.7043 | 17.648 | 11.1516 | 1.767 | 1.9642 | 1.5826 | 0.3701 | 0.5857 | 1.1165 | 0.4159 | 0.7305 | 0.4616 | 1.8629 | 1.1772 |
| native 37 | 9903 | 601 | 1218 | 849 | 645 | 260 | 216 | 386 | 1093 | 16.4775 | 15.3535 | 11.6643 | 1.4126 | 1.8884 | 1.3163 | 0.3062 | 0.4031 | 1.0732 | 0.3349 | 0.5984 | 0.4547 | 1.6946 | 1.2874 |
| native 38 | 7636 | 319 | 857 | 536 | 353 | 178 | 119 | 244 | 974 | 23.9373 | 21.6317 | 14.2463 | 1.6803 | 2.4278 | 1.5184 | 0.3321 | 0.5042 | 1.1066 | 0.3371 | 0.6912 | 0.4552 | 2.7592 | 1.8172 |
